# Immature neutrophil response is associated with cardiac complications after non-traumatic subarachnoid hemorrhage

**DOI:** 10.1007/s10238-026-02262-9

**Published:** 2026-07-22

**Authors:** Madeleine M. Puissant, Joanne T. deKay, Elizabeth K. Glisic, Joshua P. Passarelli, Angela M Leclerc, Christine Lord, Meghan Searight, Mary S. Weatherbee, Lacey Knudsen, Richard R. Riker, Teresa L. May, David Gagnon, Sergey Ryzhov, David B. Seder

**Affiliations:** 1 Department of Emergency Medicine, Maine Medical Center, 22 Bramhall St, Portland, Maine 04102 USA; 2 MaineHealth Institute for Research, Scarborough, ME 04074 USA; 3 Department of Pharmacy, Maine Medical Center, Portland, ME 04102 USA; 4 Department of Critical Care Services, Maine Medical Center, Portland, ME 04102 USA; 5 Tufts University School of Medicine, Boston, MA 02111 USA; 6 Graduate School of Biomedical Science and Engineering, University of Maine, Orono, ME 04469 USA

**Keywords:** Non-traumatic Subarachnoid Hemorrhage, Stress Cardiomyopathy, Critical Care, Cardiac Biomarkers, Systemic Inflammation, Neutrophils, Cardiac Abnormalities

## Abstract

Acute non-traumatic subarachnoid hemorrhage (SAH) is complicated by systemic inflammation and cardiac dysfunction that contribute to the high rates of morbidity and mortality. To better define the mechanistic relationship between immune response and cardiac complications, we evaluated subpopulations of circulating immune cells and their associations with abnormalities of cardiac rhythm, injury biomarkers, and mechanical function after hemorrhage. In this single-site prospective observational study, we screened all adult patients presenting to our neurocritical care unit with non-traumatic SAH who required external ventricular drain (EVD) placement for treatment of hydrocephalus. Following informed consent, peripheral blood samples were obtained at post-bleed days (PBDs) 1, 3, 5, 7, and 10. Subjects were separated into groups based on evidence of cardiac abnormalities (cAbn(+))” versus “no cardiac abnormalities (cAbn(-)),” and flow cytometric analysis was used to identify and quantify immune cell subpopulations. Circulating neutrophil-secreted factors, including myeloperoxidase (MPO), were measured in plasma using ELISA. We measured associations between these immune factors and cardiac abnormalities. Sixty patients were enrolled between January 2018 and August 2023. Our patient cohort was 65% female with a median age of 58 (IQR 51–68). Poor grade SAH (Hunt Hess = 4–5) occurred in 52% (*n* = 31) of patients. In the overall cohort 47 (78%) required prolonged mechanical ventilation (> 24-hours), and 52 (87%) underwent coil embolization/clipping of their aneurysm. The average ICU and hospital lengths of stay (LOS) were 18.5 (IQR 12.2–22) and 23 (IQR 18.2–33.8) days, respectively. In-hospital mortality for enrolled patients was 11.8% and overall mortality 6 months after discharge was 25% (*n* = 15). 40% (*n* = 24) of patients were discharged home, 37% (*n* = 22) to acute rehabilitation, 8% (*n* = 5) to SNF, and 3% (*n* = 2) to hospice. Cardiac abnormalities after SAH were associated with increased circulating neutrophil counts but not monocyte or lymphocyte levels. Circulating neutrophils in patients with cardiac abnormalities also had reduced cell-surface expression of maturation markers CD10, CD14, and CD16 in patients with cardiac abnormalities. Elevated levels of immature neutrophils and circulating levels of lipocalin-2, and myeloperoxidase (MPO) correlated with increased Hunt-Hess score, ICU, and hospital length of stay. Higher grade SAH was associated with increased immature neutrophil response, which was associated with cardiac abnormalities. These cardiac abnormalities tracked with increased levels of pro-inflammatory lipocalin-2 and MPO, and worse outcomes.

**Supplementary Information:**

The online version contains supplementary material available at https://doi.org/10.1007/s10238-026-02262-9.

## Introduction

Non-traumatic subarachnoid hemorrhage (SAH) accounts for approximately 30% of all hemorrhagic strokes and affects a younger patient population compared to other stroke types. In the US, the annual incidence is estimated at 10–14 per 100,000 individuals and results in a high burden of morbidity and mortality, despite advances in diagnosis and treatment [1–5]. Cardiac effects after SAH include structural and electrical changes in the heart, which are associated with higher rates of in-hospital complications and worse neurological outcomes [6,7]. These cardiovascular effects are thought to result in part from an acute hemorrhage-induced surge in catecholamines, which acts on adrenergic receptors on almost all peripheral tissue, including the myocardium, leading to structural, electrical, and functional alterations [8–11].

Catecholamine-induced changes are evident immediately after hemorrhage, most commonly: electrocardiogram (ECG) abnormalities including tachyarrhythmias, ST-segment changes, T-wave inversion, and QTc prolongation [12–14]. Some patients also develop echocardiographic evidence of regional wall motion abnormalities or global left ventricular dysfunction, sometimes accompanied by hemodynamic instability and elevated cardiac biomarkers consistent with myocardial injury [15–17]. These cardiac effects range from mild, clinically insignificant findings to severe circulatory compromise requiring inotropic or mechanical support, which can hinder neurologic recovery and contribute to secondary brain injury, or cause death [18–20].

Exaggerated inflammatory response is a prognostic factor after SAH and secondarily affects other organs, including the brain and heart [21,22]. In animal models, SAH has been shown to increase astrocyte and microglial activity, promote inflammatory cell infiltration of the central nervous system (CNS), and up-regulate cytokine release and inflammatory factor expression in brain tissue- processes thought to exacerbate neurologic injury and worsen long-term neurologic outcomes [11,14,23]. Inflammatory effects on the cardiovascular system, including infiltration of the vascular endothelium and myocardium, lead to vascular leakage, dysfunctional vascular tone, dysrhythmias, ischemia, and injury [18,24,25].

Recent studies highlight the critical role of specific innate immune cells, neutrophils, which are thought to be critical drivers of the early systemic and CNS inflammatory response after neurologic injury [26,27]. Activated neutrophils cross the blood-brain barrier and infiltrate the brain parenchyma, leading to edema, necrosis, and scar formation [28,29]. They also infiltrate myocardial tissue, affecting both dromotropic properties (speed of electrical impulses through the heart; predisposing to arrhythmias, including QT interval elongation) and ionotropic function (promoting cardiac ischemia and contraction band necrosis) [30,31]. Neutrophilia has been associated with increased hospital mortality after SAH [32] Neutrophils can be further divided into subsets that differ in maturation state and functional properties [33,34]. However, the role of neutrophil subsets in SAH has not yet been investigated. The goal of this study was to characterize the association in subsets of immature and mature neutrophils with cardiac abnormalities and outcomes after SAH.

## Methods

### Patient population and study design

In this single-site prospective observational study, we screened all adult patients presenting to our Neurocritical Care unit between September 2017 and August 2023, with non-traumatic SAH and EVD placement. Our neurocritical care unit serves a broad and predominantly rural catchment area and admits approximately 80 patients with acute non-traumatic SAH annually. Patients were excluded from the study if they were pregnant, less than 18 years of age, had a traumatic SAH, had delayed hospital presentation, or the patient/LAR declined participation. After written informed consent was obtained from patient/LAR, peripheral blood was obtained on post-bleed days (PBDs) 1, 3, 5, 7, and 10. In addition to biospecimen collection, electronic medical record review of the hospital course was used to acquire demographic and clinical data. Clinical data from hospital courses included pre-morbid comorbidities and medications, electrocardiograms (ECGs), echocardiograms, hospital-acquired lab tests, complications during hospital course, interventions (including neurosurgical procedures, intubation, etc.), infections and antibiotic use, vasopressor administration, intensive care unit (ICU), and hospital length of stay (LOS, days), and hospital mortality. Our local Internal Review Board reviewed and approved this study under MaineHealth IRB#1,100,316; this study was conducted in accordance with the Declaration of Helsinki and adhered to the core ethical principles for medical research involving human subjects including prioritizing participant welfare, informed consent, and independent ethical review. Informed consent was obtained from all individual participants or their legally authorized representative (LAR) in the study.

### Clinical scoring of cardiac abnormalities after SAH

Severity of cardiac stress after SAH in this cohort was assessed through evaluation of ECG, echocardiograms, and cardiac biomarkers within the first 5 days of hospital course. We developed a scoring system with one point each assigned for: elongated QTc (>440 for males, >460 for females) on ECG, elevated troponin T level (ELISA), and abnormal echocardiogram findings for a cumulative score ranging from 0 to 3. Echocardiograms were designated as “abnormal” if they exhibited new wall motion abnormalities or new global hypokinesis with or without change to left ventricular ejection fraction (LVEF) that returned to normal on follow-up echocardiograms that were obtained later in the hospital course. Echocardiograms were obtained on patients per discretion of clinical team during hospitalization which occurred in 48 of 60 patients (80%). These scores were dichotomized into “no cardiac abnormalities (cAbn(-); score of 0)” versus “cardiac abnormalities (cAbn(+), scores ≥1 Table 1; with expanded details in **Supplemental Table ****1**).” A total of 34 patients were considered positive for cardiac abnormalities (score between 1 and 3).

**Table 1:** Cardiac abnormalities *n*=60 stratified by the presence (cABn+) or absence (CAbn-) of cardiac abnormalities including elongation of QT-segment above sex maximum on ECG, elevation of the cardiac biomarker, troponin, and abnormal echocardiogram findings

	cAbn(+)	cAbn(-)
	Total *n* (%)	Total *n* (%)
QTc Elongation	8 (24)	0 (0)
Troponin Elevation	28 (82)	0 (0)
Abnormal Echo	11 (32)	0 (0)

### Blood sample collection

Whole blood (8.5mL), collected using BD Vacutainer ACD tubes, was processed as previously described [35]. In brief, blood aliquots (50µL/test) were taken for flow cytometric analysis and determination of white blood cell (WBC) count. Platelet-free plasma (PFP) was prepared by consecutive centrifugations at 2,000 g for 20 min at room temperature. PFP samples were stored at -80 °C until further analysis. No more than one freeze/thaw cycle was permitted for PFP samples to avoid protein degradation.

### Flow cytometric analysis

Blood aliquots (50µL) underwent erythrocyte lysis with ammonium chloride lysing solution (150 mmol/L NH_4_Cl, 10 mmol/L NaHCO_3_, and 1 mmol/L EDTA, pH 7.4). WBC count was determined manually with Trypan Blue and a hemocytometer. WBCs (10^6^/mL) were treated with whole-molecule mouse and human IgGs to prevent nonspecific binding, followed by incubation with the antibodies noted below for 25 min at 4 °C. Cells were washed once with 10 volumes of cold PBS/0.5% BSA/2mM EDTA before data acquisition. The following antibodies were used: CD3 (clone UCHT1), CD10 (clone HI10a), CD14 (clone HCD14), CD16 (clone 3G8), CD19 (clone HIB19), CD45 (clone H130), and HLA-DR (clone L243) (all obtained from BioLegend). The number of cells in subpopulations was determined using the total WBC count and the percentage of cells in each subpopulation. All antibodies were titrated to achieve a clear separation between positive and negative cell populations while maintaining a low background. Isotype-matched control antibodies were used to determine the level of undesired non-specific binding. Viable cells vs. nonviable cells/cell debris were distinguished using DAPI (to detect dead nucleated cells). Antigen negativity was defined as having the same fluorescent intensity as the isotype control.

Intracellular staining was performed as previously described with modifications [36]. WBC were incubated with LIVE/DEAD^®^ Fixable Violet Stain kit (VioDye) for the detection of non-nucleated cell debris (Life Technologies, Carlsbad, CA), following staining with antibodies against cell surface CD10 (clone HI10a), CD14 (clone HCD14), CD16 (clone 3G8), and CD66b (clone 6/40c) (all obtained from BioLegend). Cells were then fixed and permeabilized using BD Cytofix/Cytoperm™ (BD Biosciences). FITC-conjugated anti-MPO antibody (MPO421-8B2, BioLegend) and IgG controls (MOPC-21) were used to determine intracellular levels of MPO in CD10^−^ and CD10^+^ subsets of CD66b-expressing neutrophils.

Serial blood draw to determine initial differences in the total number of white blood cells, neutrophils, monocytes, and lymphocytes was obtained from 28 subjects on PBD1 (54% missing), 57 subjects on PBD3 (5% missing), 58 subjects on PBD5 and PBD10 (3% missing) and 59 subjects on PBD7 (2% missing). Missing data was attributed to enrollment after PBD1 collection window and inability to collect samples during study window due to patient undergoing procedures or lab closure. The subpopulations of neutrophils, monocytes, lymphocytes, immature CD16low/CD14low neutrophils, and mature CD16highCD14high -expressing neutrophils were analyzed in blood samples obtained from 57 patients on post-bleeding day 3 (95%, 2 collections were missed, 1 sample was frozen at the hospital). The subset of CD10 expressing neutrophils was determined in the last 26 patients enrolled in the study.

Data acquisition was performed on a MACSQuant Analyzer 10 (Miltenyi Biotec, Inc). Data were analyzed using WinList 5.0 and FlowJo 10.

### Circulating factors quantification

Circulating Lipocalin-2, MPO, NT proBNP, and Troponin I protein levels were determined using Human Lipocalin-2/NGAL, Myeloperoxidase, NT proBNP, and Troponin I/TNNI3 Duoset ELISA (Bio-Techne/R&D Systems) kits. Troponin T levels were measured with RayBiotech’s Human Troponin T ELISA kit.

### Statistical analysis

The Kolmogorov-Smirnov test was used for the determination of normality. Skewed data are presented as median and interquartile range. Comparisons between two groups were performed using the Mann-Whitney test. Comparisons between three groups or more were performed using ordinary one-way ANOVA with Kruskal-Wallis test with Dunn’s multiple comparison test or mixed-effects analysis with Tukey’s multiple-comparisons post-test. Correlation analyses were performed using the Spearman correlation coefficient. A p-value less than 0.05 was considered statistically significant. A modified Poisson generalized regression was used to adjust for factors that may impact granulopoiesis and increase the number of immature neutrophils. Those factors included age, Hunt and Hess score, vasopressor exposure, infection, and mechanical ventilation. Statistical analyses were performed using SPSS Statistics v32 software package.

## Results

### Patient demographics and characteristics

**Fig. 1:**
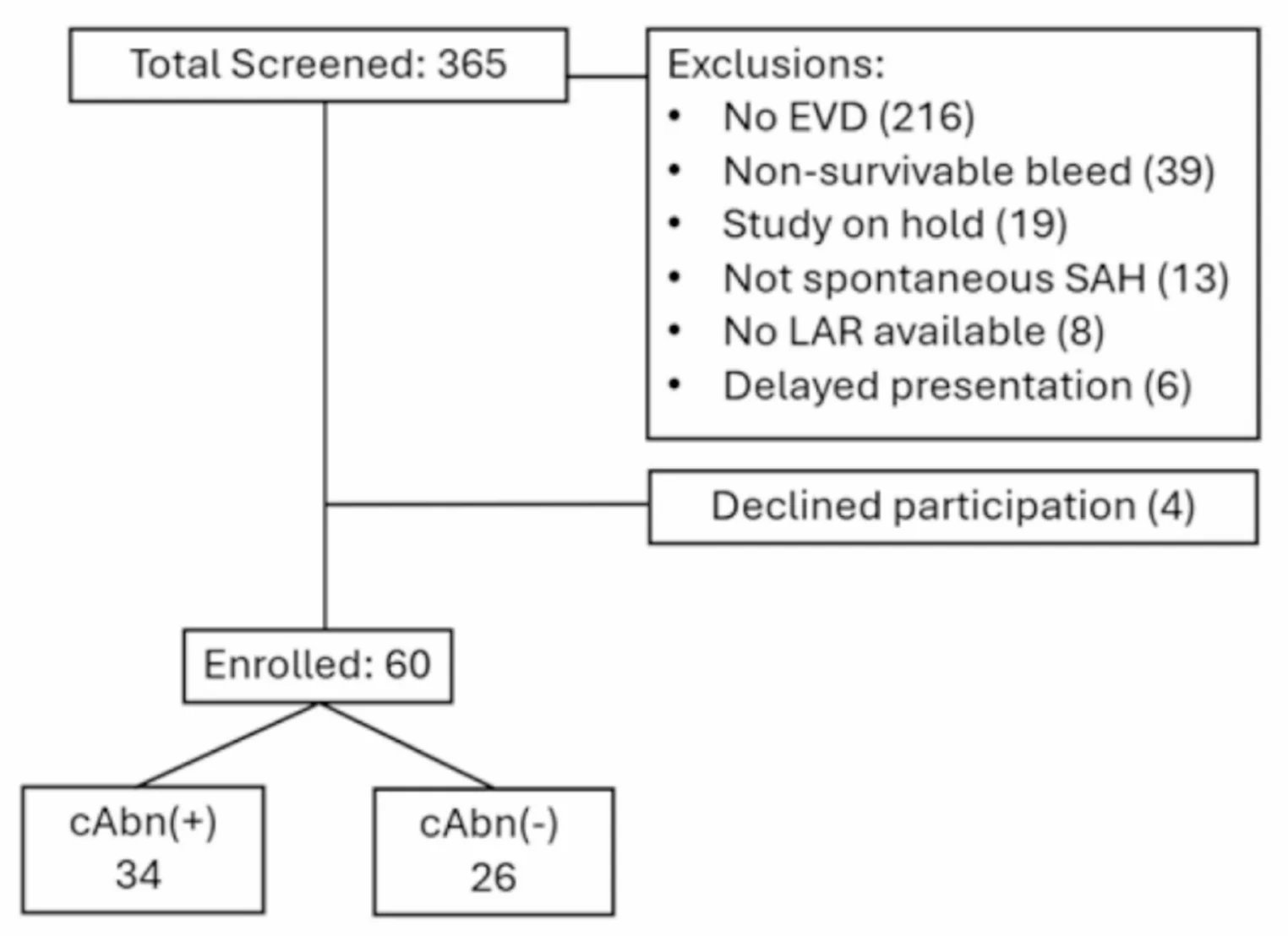
Study enrollment. Subarachnoid hemorrhage (SAH); external ventricular device (EVD); Do Not Resuscitate (DNR); Legally Authorized Representative (LAR)

We identified a total of 149 SAH patients with EVD over the study period; after exclusions, 60 were enrolled and included in the analysis (Fig. 1). Our cohort was 65% female with median age of 58 (IQR 51, 68). 51.7% (*n* = 31) of patients had poor grade SAH (Hunt Hess (HH) score of 4–5). Five patients (8%) had a prior history of CAD; hypertension (*n* = 40, 67%) and hyperlipidemia (*n* = 18, 30%) were high (Table 2 ). Slightly more than half of the patients (*n* = 34, 56%) had evidence of cardiac abnormalities, defined as elongated QTc, abnormal troponin level, and/or abnormal echocardiogram (Table 1).

**Table 2:**
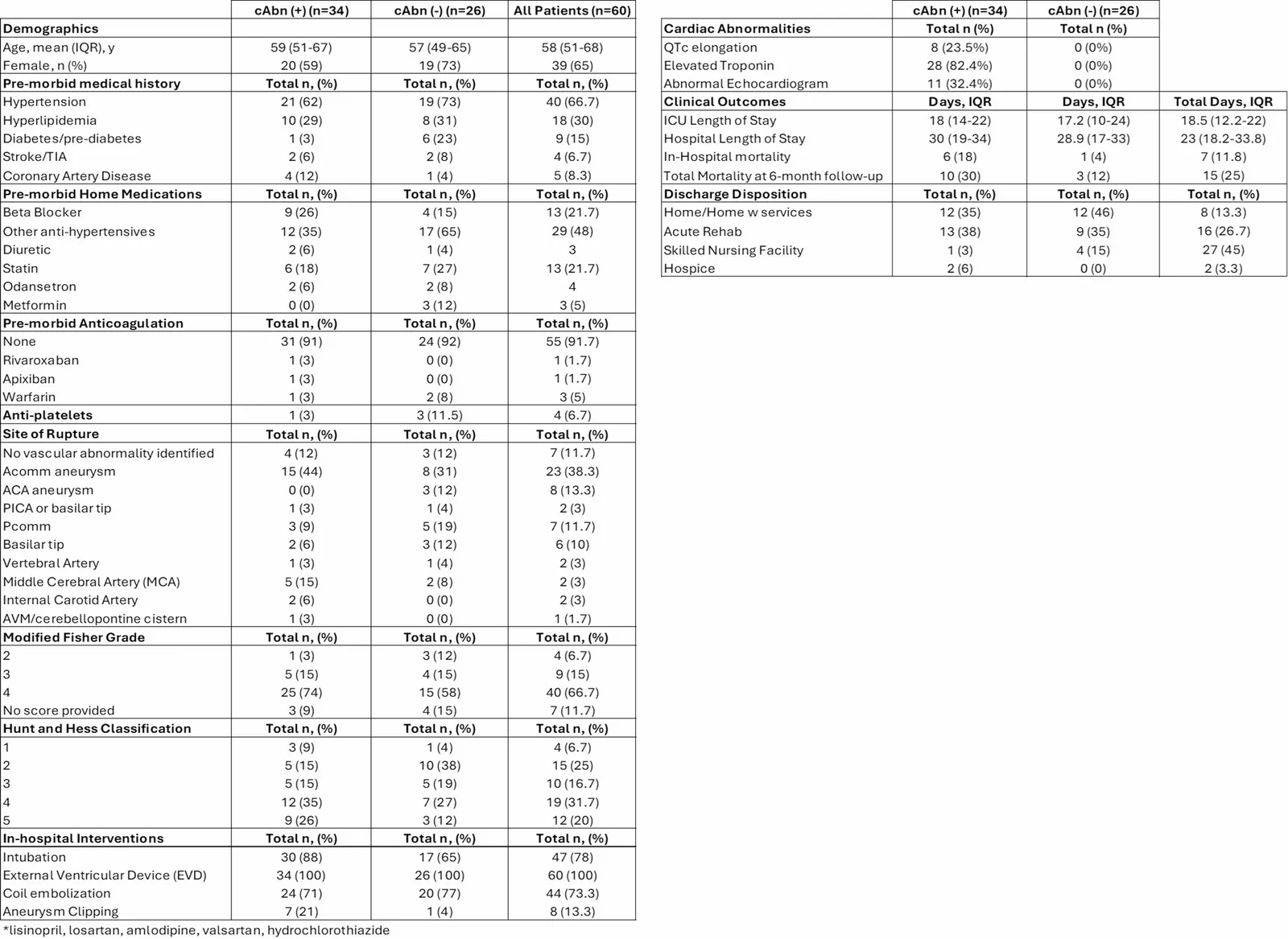
Subarachnoid hemorrhages (SAH) patient demographics, *n*=60 stratified by the presence (cABn+) or absence (cAbn-) of cardiac abnormalities

### Hospital Course

A majority of this cohort required prolonged mechanical ventilation (> 24-hour (IQR 12.2–22) and 23 (IQR 18.2–33.8) days, respectively) during hospital course (*n* = 47; 78%), and 52 (87%) underwent coil embolization/clipping of their aneurysm. The majority of study participants received antibiotics within the first 10-days of hospitalization, most commonly as surgical prophylaxis but also in treatment of presumed or culture proven infections (Table 3). The median neurocritical care unit and hospital length of stay (LOS) was 19. In-hospital mortality for enrolled patients was 12% and total mortality at 6-month follow-up was 25% (*n* = 15). Twenty-four patients (40%) were discharged home, 27 (45%) were discharged to skilled nursing facilities (SNFs), and 2 (3%) were discharged to hospice (Table 2 , see above).

**Table 3:**
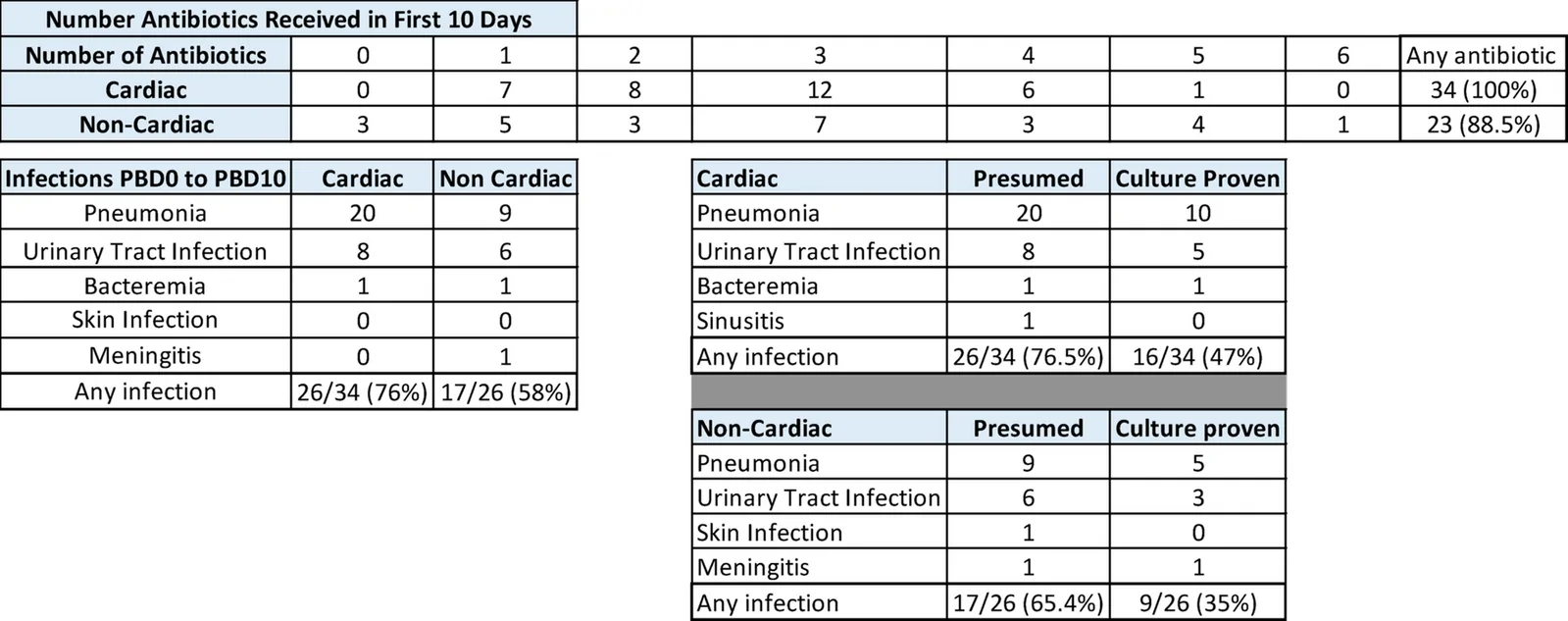
Comparison of the number of antibiotics administered, presumed infections, and culture proven infections in the first 10-days of hospitalization after SAH in cardiac and non-cardiac cohorts

### Cardiac stress was associated with an early increase in the number of circulating neutrophils

**Fig. 2:**
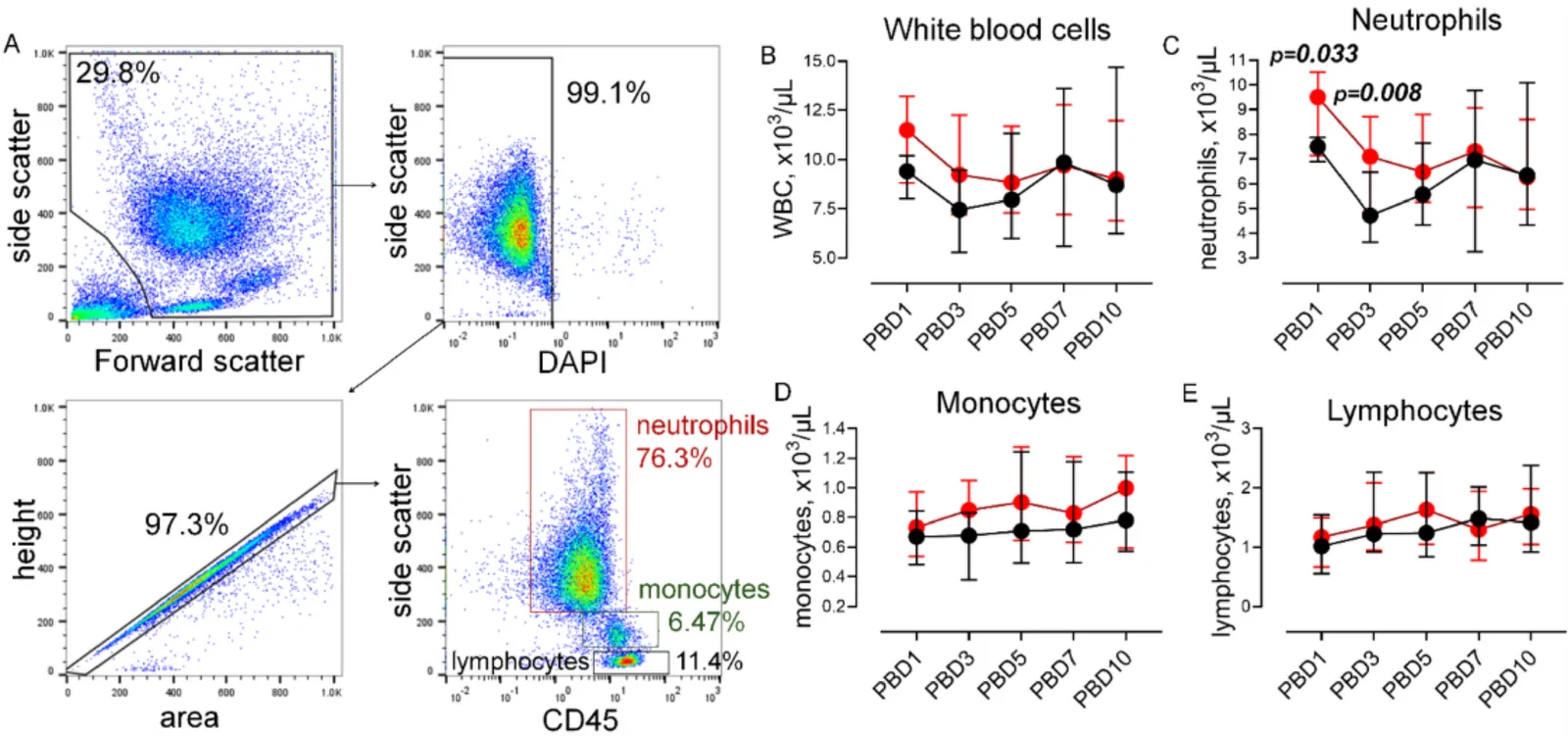
Neutrophil counts are elevated early after SAH in subjects with cardiac complications. Subjects with SAH were divided into two major groups, clinically defined as subjects with or without cardiac abnormalities as described in Methods. **A**. Flow cytometric analysis was performed after red blood lysis. The unlysed erythrocytes and platelets were identified as SSC^low^/FSC^low^ and excluded from the analysis, as shown in the upper-left plot. Viable cells (upper right plot) and singlets (lower left plot) were used to determine neutrophils defined as SSC^high^/CD45^low^ (red gate), SSC^intermediate^/CD45^intermediate^ monocytes (green gate), and SSC^low^/CD45^high^ lymphocytes (black gate) as shown in the lower right plot. **B-E**. Graphical representation of flow cytometric data showing the number of (**B**) CD45 + white blood cells, (**C**) neutrophils, (**D**) monocytes, and (**E**) lymphocytes in SAH subjects without (*n* = 26, black) and with cardiac abnormalities (*n* = 34, red) on different post-bleed days (PBD). Mixed-effects model, Tukey’s post-test

After identifying patients in our cohort with evidence of cardiac abnormalities after hemorrhage, we sought to further characterize the subpopulations of immune cells that may be significant contributors to this pathological process. Figure 2A shows a flow cytometric strategy to examine major subpopulations of circulating white blood cells using side-scatter characteristics and expression of pan-leukocyte marker CD45. All analyses were performed after red blood cell lysis, using DAPI to distinguish dead versus viable cells and height-to-area forward scatter to exclude cell aggregates from the analysis. As shown in Fig. 2B-E, only circulating neutrophils, but not monocytes or lymphocytes, significantly increased on PBD1 (neutrophil median 7.5 × 10^3^/µL cAbn(-) vs. 9.5 × 10^3^/µL cAbn(+)) and PBD3 (4.7 × 10^3^/µL cAbn(-) vs. 7.1 × 10^3^/µL cAbn(+)) in subjects with evidence of cardiac stress.

### The number of circulating neutrophils was associated with the length of the QTc interval

**Fig. 3:**
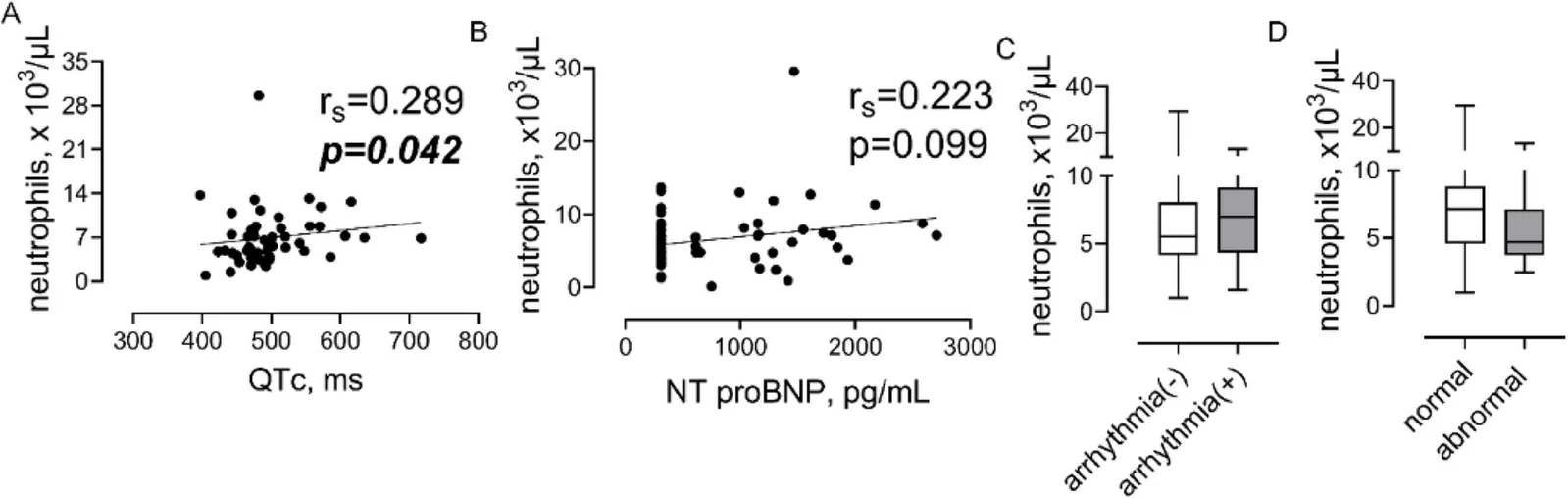
The number of circulating neutrophils is positively associated with the length of QTc intervals. Analysis of neutrophils, QTc, N-terminal pro B-type natriuretic peptide (NT-pro-BNP), cardiac rhythm, and function was performed on PBD3. **A-B**. Spearman correlation between the number of neutrophils and (**A**) QTc, and (**B**) N-terminal pro B-type natriuretic peptide (NT-pro-BNP); *n* = 60. **C**-**D**. Number of neutrophils in subjects with (**C**) normal cardiac rhythm (*arrhythmia(-)*, *n* = 41) and arrhythmia (*arrhythmia(+)*, *n* = 14), and (**D**) normal (*n* = 31) and abnormal (*n* = 14) cardiac function examined by echocardiography. Mann-Whitney test

To determine the contribution of neutrophils to specific cardiac complications, we analyzed the association between neutrophil count on PBD3 and cardiac outcomes. The analysis revealed a positive correlation between absolute neutrophil count and QTc length on ECG after SAH (Fig. 3A). Although not statistically significant, our data also indicates numerical association between neutrophils and NT-proBNP (Fig. 3B). No associations were found between total neutrophils and arrhythmias (Fig. 3C) or abnormalities seen on an echocardiogram.

### Decreased expression of CD14 and CD16, and an elevated number of CD10^−^ immature neutrophils, are associated with the elevated length of QTc intervals and poor outcomes after SAH

**Fig. 4:**
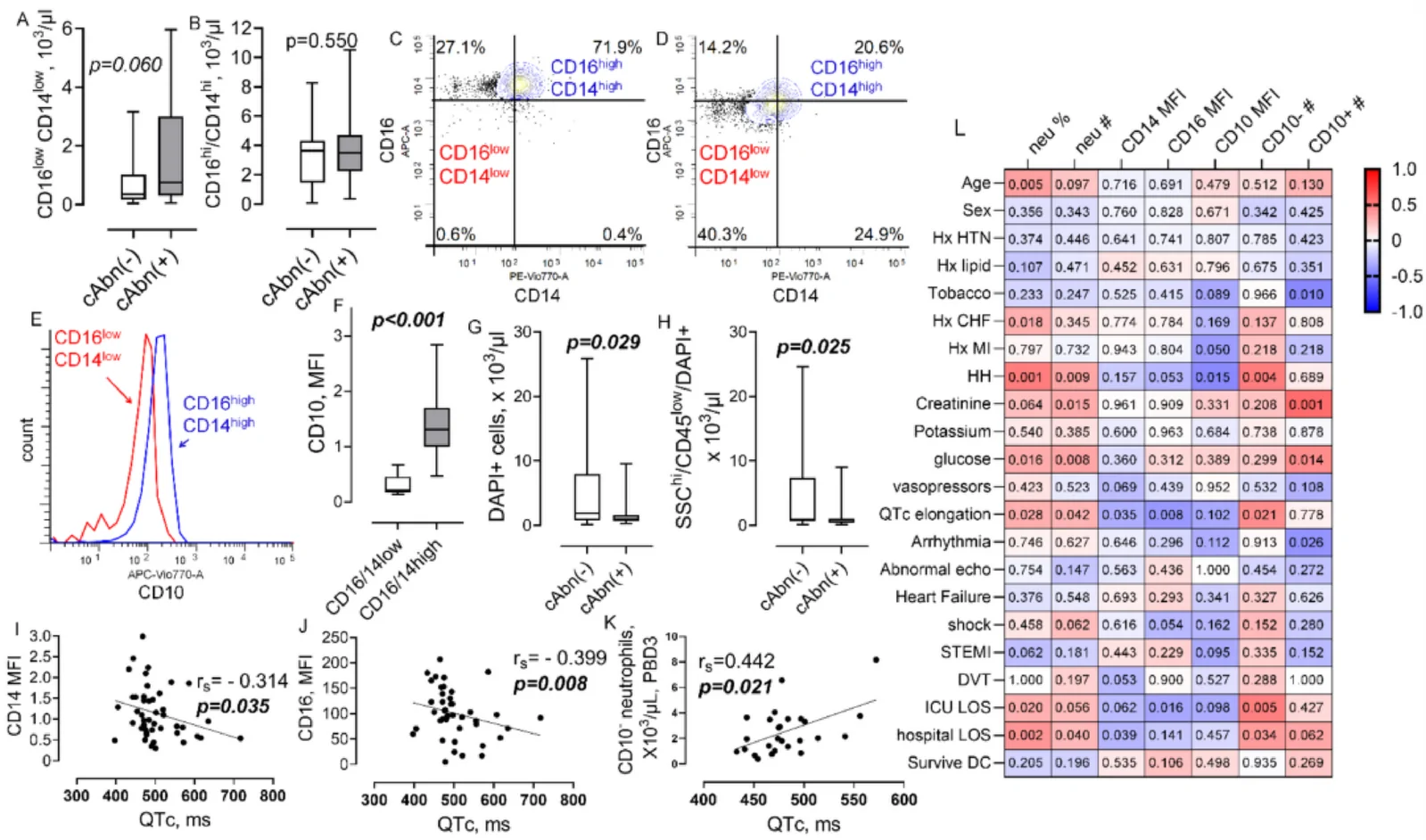
Association of mature and immature subsets of neutrophils with clinical cardiac stress and outcomes after SAH. Analysis was performed on PBD3. **A-B**. Number of (**A**) CD16^low^/CD14^low^ and (**B**) CD16^hi^/CD14^hi^ neutrophils in subjects without (cAbn(-), *n* = 26) and with (cAbn(+), *n* = 34) cardiac complications; Mann-Whitney test. **C**-**D**. Representative flow cytometric plots showing the percentage of CD16^low^/CD14^low^ (lower left quadrant) and CD16^high^/CD14^high^ (upper right quadrant) neutrophils in subjects with (**C**) low QTc length (423 ms, left plot) and (**D**) high QTc length (572 ms, right plot). **E**. Representative histograms showing expression of CD10 on CD16^low^/CD14^low^ (red) and CD16^high^/CD14^high^ (blue) neutrophils. **F**. Graphical representation of mean fluorescence intensity of cell-surface CD10 expression on CD16^low^/CD14^low^ (red) and CD16^high^/CD14^high^ (blue) neutrophils; *n* = 26, Mann-Whitney test. **G**-**H**. Number of (**G**) DAPI-positive dead cells, and (**H**) DAPI-positive dead neutrophils, defined as SSC^high^/CD45^low^/DAPI + in subjects without (cAbn(-), *n* = 26) and with (cAbn(+), *n* = 34) cardiac complications; Mann-Whitney test. **I**-**J**. Spearman correlation analysis of associations between levels of cell surface (**I**) CD14, and (**J**) CD16 with length of QTc intervals; *n* = 60. K. Spearman correlation analysis of associations between the number of CD10^−^ immature neutrophils and the length of QTc intervals; *n* = 26. **L**. Correlogram showing p-values of Spearman correlation analysis between subsets of mature and immature neutrophils and clinical parameters of subjects with SAH on PBD3, *n* = 60. Spearman correlation. *P* values are indicated in the boxes; color keys denote coefficients. *DVT*, deep vein thrombosis; *HH*, Hunt–Hess scores; *Hx*, history; *ICU*, intensive care unit; *LOS*, length of stay; *MI*, myocardial infarction; *Survive DC*, survival at hospital discharge

To determine if the increased number of neutrophils is also associated with their activation, we examined levels of cell-surface expression of CD14, a co-receptor for toll-like receptors, and CD16, also known as Fcg receptor III. Our analysis revealed a trend toward accumulation of neutrophils with low expression of both proteins in subjects with cardiac abnormalities compared to the non-cardiac group (Fig. 4A). No differences were found in the number of CD16^high^/CD14^high^ neutrophils between the two groups (Fig. 4B).

CD14 and CD16 are also associated with neutrophil maturation, and lower levels of these markers characterize immature neutrophils. Additionally, we analyzed cell-surface expression of CD10, a zinc-dependent metalloprotease enzyme, which is strongly associated with maturation, using flow cytometry in the last 26 patients enrolled in the study. We then studied CD10 expression in neutrophil subsets using a strategy illustrated in Fig. 4C-D. As shown in Fig. 4E and **F**, the expression of CD10 was significantly lower in CD16^low^/CD14^low^ compared to CD16^high^/CD14^high^ neutrophils, indicating a lower maturation status of neutrophils with decreased cell surface expression of CD14 and CD16. The level of CD16 can also be reduced on neutrophils undergoing apoptosis. However, our data indicated a reduced total number of DAPI-positive events and DAPI-positive neutrophils, defined as SSC^high^/CD45^low^ cells **(**Figs. 4G and **H**) in subjects with cardiac stress compared to those without cardiac complications.

The levels of cell-surface expressions of CD14 and CD16 were inversely correlated with the QTc interval (Fig. 4I and **J**), indicating the potential role of less mature neutrophils in cardiac rhythm disturbances. In agreement with this, we also found a positive, moderate association between the number of CD10^−^ immature neutrophils and the length of QTc intervals (Fig. 4K).

We also examined the relationship between neutrophils and established clinical markers of disease severity and outcome after SAH (Fig. 4L). Higher relative and absolute neutrophil counts, measured on PBD 3, were associated with more severe hemorrhage, as reflected by higher Hunt–Hess scores, longer ICU and hospital length of stay. Similar associations were observed for immature neutrophils, as indicated by a positive association between CD10^−^neutrophils and inverse correlations with neutrophils expressing lower levels of CD16 and CD10 on their surface. No associations were found between CD10^+^ mature neutrophils and HH scores, or ICU and hospital LOS. However, the number of CD10^+^ neutrophils was inversely correlated with arrhythmias, indicating a potential protective cardiac effect of mature neutrophils.

Even after adjusting for age, Hunt & Hess score (disease severity), vasopressor exposure, infection, and mechanical infection there remained a ~10-15% increase in risk of cardiac abnormalities in patients with a higher number of CD16low/CD14low neutrophils (Table 4).

**Table 4:**
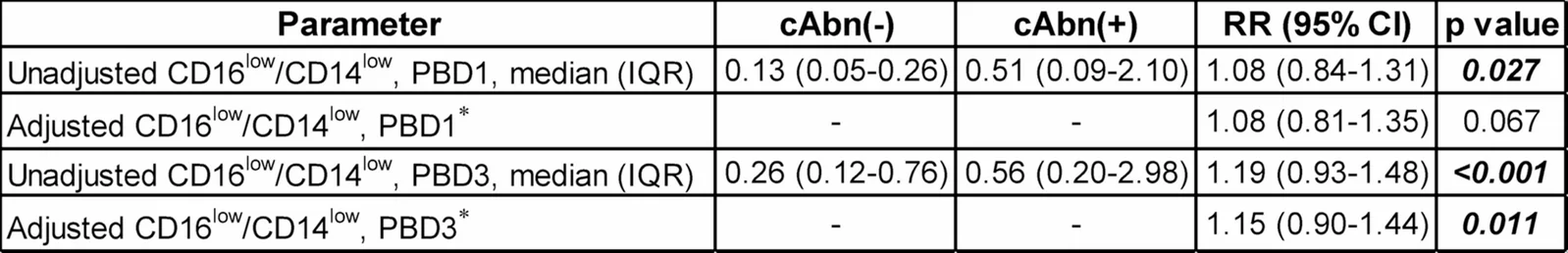
Relative risk (RR) of a higher subset of CD16^low^/CD14^low^ neutrophils and clinical cardiac stress. ^∗^Adjusted for age, Hunt and Hess score, vasopressor exposure, infection, and mechanical ventilation. See supplemental tables for additional analyses

### Levels of pro-inflammatory lipocalin-2 and MPO are associated with a higher number of immature neutrophils and poor outcomes after SAH

**Fig. 5:**
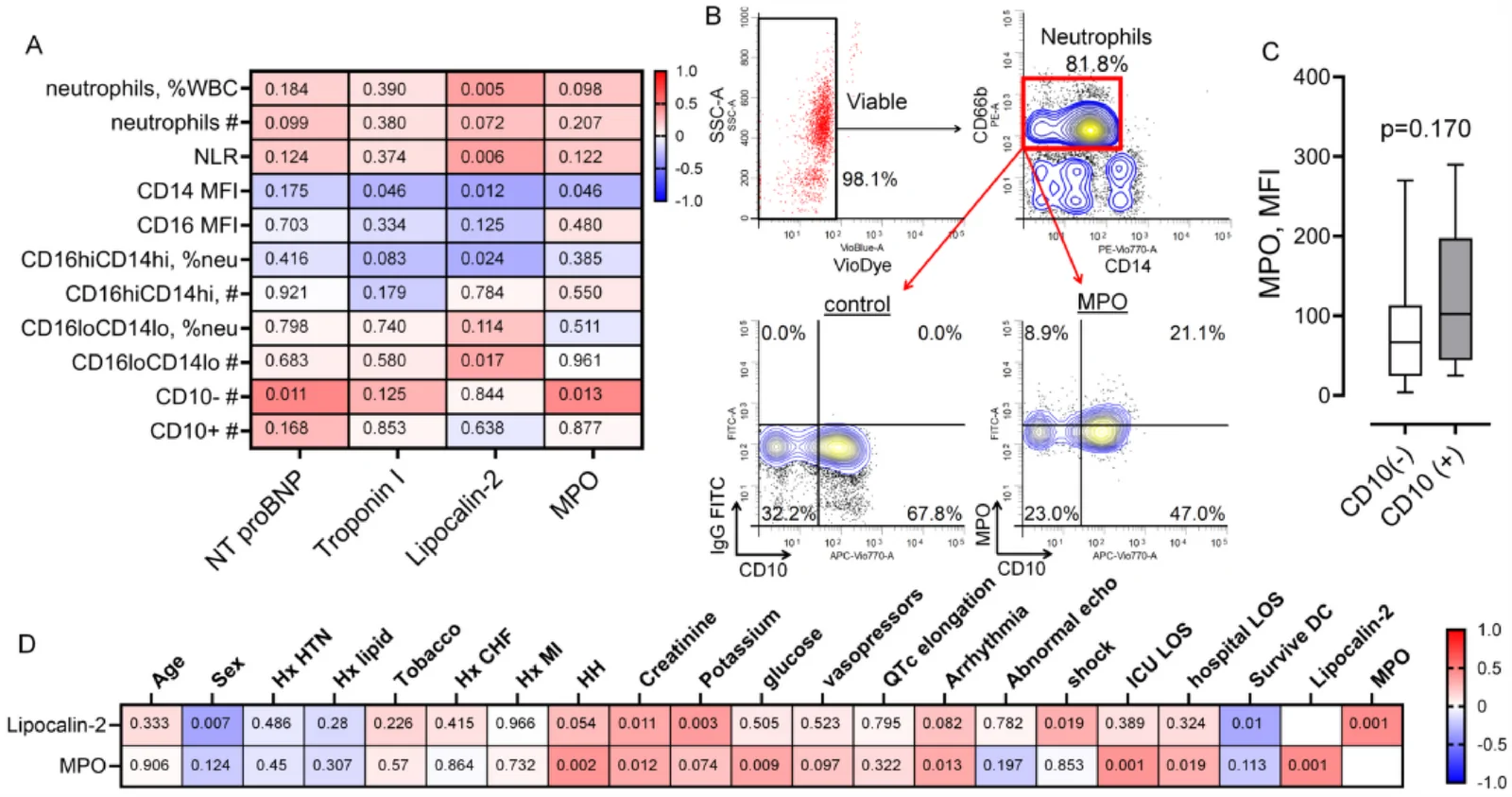
Neutrophil-secreted pro-inflammatory factors, Lipocalin-2 and MPO, are associated with cardiac complications and poor outcomes after SAH. The levels of circulating factors were measured on PBD3. **A**. A correlogram showing Spearman correlation p-values between subsets of mature and immature neutrophils and levels of circulating NT-proBNP, Troponin I, Lipocalin-2, and MPO. *MFI*, mean fluorescence intensity; *NLR*, neutrophils-to-lymphocytes ratio; *WBC*, white blood cells; *hi*, high expression; *lo*, low or no expression; #, number. **B**. Flow cytometric strategy to examine the intracellular levels of MPO in CD10^−^ immature and CD10^+^ mature neutrophils. Viable cells were distinguished using amine-reactive fixable dye (VioDye, upper left plot), and saponin A-permeabilized CD66b^+^ neutrophils (red gate, upper right plot) were used to analyze the level of MPO using IgG FITC control antibodies (lower left plot) and specific antibody against MPO (lower right plot). **C**. Graphical representation of data on the level of intracellular MPO in immature (CD10(-)) and mature (CD10(+)) neutrophils. *n* = 10, Mann-Whitney test. **D**. Associations between circulating neutrophil-secreted factors (PBD3) and outcomes, *n* = 60. Spearman correlation. *P* values are indicated in the boxes; color keys denote coefficients. *CHF*, congestive heart failure; *HH*, Hunt–Hess scores; *Hx*, history; *ICU*, intensive care unit; *LOS*, length of stay; *MI*, myocardial infarction; *Survive DC*, survival at hospital discharge

Neutrophils impact systemic and local inflammatory responses by releasing pro-inflammatory compounds stored in intracellular compartments. To determine whether neutrophil degranulation contributes to cardiac abnormalities after SAH, we analyzed circulating levels of lipocalin-2 and MPO, which are stored in distinct neutrophil granule types and released upon stimulation. The correlation analysis revealed the positive associations between the number of CD10^−^ immature neutrophils and NT-proBNP, and between CD10^−^ neutrophils and circulating MPO levels. We also found a positive association between the number of less mature CD16^low^/CD14^low^ neutrophils and lipocalin-2. In contrast, more mature CD16^high^/CD14^high^ neutrophils were inversely correlated with lipocalin-2 (Fig. 5A).

To determine if the positive correlation between CD10^−^ neutrophils and circulating MPO levels is the result of higher generation of MPO in immature neutrophils, we performed an analysis of the intracellular level of MPO in neutrophils freshly isolated from blood. This analysis was conducted in a subset of ten SAH subjects, who were most recently enrolled in the study. We used the neutrophil cell-surface marker CD66b to distinguish between neutrophils and monocytes, which also produce MPO. As shown in Fig. 5B, the intracellular levels of MPO were high in approximately one-third of each subset, CD10^−^ immature and CD10^+^ mature neutrophils. Analysis of mean fluorescence intensity, which indicates the total level of MPO generation, also demonstrated no differences between CD10^−^ and CD10^+^ neutrophils (Fig. 5C).

Correlation analysis revealed positive associations between lipocalin-2 and MPO, creatinine level, electrolyte imbalances, and HH scores (Fig. 5D). In addition, a high circulating level of MPO was also associated with arrhythmia, increased ICU and hospital length of stay; subjects with a high level of lipocalin-2 were characterized by a lower survival rate after SAH.

## Discussion

In this prospective study, we show an association between early elevation of immature neutrophils and cardiac stress after SAH, suggesting granulocytes could be a mechanistic link between neurologic injury and cardiac complications. The results support a model in which SAH induces mobilization of CD10^−^ immature neutrophils may contribute to cardiac disturbances via the release of pro-inflammatory lipocalin-2 and MPO, which is associated with worse clinical outcomes.

We observed that patients with evidence of cardiac stress after SAH have increased circulating neutrophils compared to those without cardiac abnormalities. This supports prior work demonstrating that SAH induced a robust systemic inflammatory response and extends these observations by implicating neutrophil predominance as a potential mediator of extracerebral organ injury [11,21,23,37]. The shift toward neutrophil dominance may reflect both stress leukocytosis and targeted mobilization of myeloid cells in response to sympathetic nervous system activation triggered by aneurysm rupture, or by acute non-aneurysmal hemorrhage [12,27].

We found that higher neutrophil counts were positively associated with prolongation of QTc interval on ECG. While autonomic dysregulation has traditionally been emphasized in this setting, our results suggest that neutrophils may also contribute to cardiac rhythm abnormalities. In addition to their association with QTc interval length, neutrophil levels also trended with elevated NT-proBNP levels, a marker of myocardial wall stress, as well as with the occurrence of clinically documented arrhythmias during hospitalization. These findings suggest that neutrophil mediated inflammation may influence both the structural and electrophysiologic manifestations of cardiac stress changes in the heart after SAH. Notably, we did not observe associations between neutrophil counts and echocardiographic parameters of myocardial function, which may reflect the multifactorial nature of cardiac stress in SAH, small size of our sample, and the limitations of echocardiography in detecting subtle or transient myocardial injury in this patient population.

Recent studies have revealed the heterogeneity of circulating neutrophils [33,34]. Mature cells represent the vast majority of neutrophils, with a marginal fraction of immature neutrophils at baseline [38,39]. However, immature neutrophils can be rapidly mobilized from the bone marrow under stress, mediated in part by sympathetic activation. Immature neutrophils are characterized by lower or no expression of CD10, CD14, and CD16, depending on maturation stage [40,41]. This study demonstrated a positive association between CD10^−^ immature neutrophils with decreased expression of CD14 and CD16, not CD10^+^ mature neutrophils, and with QTc prolongation. Higher immature neutrophils were also associated with worse outcomes, supporting the notion that severe SAH may induce an emergency granulopoiesis with detrimental systemic consequences. Although we cannot exclude the contribution of mature neutrophils to SAH-induced pathology, the findings in this study suggest that both the number and the immaturity of neutrophils may contribute to cardiac stress and dysfunction.

Our data show that systemic neutrophil degranulation is associated with cardiac complications and worse functional outcomes. Neutrophil degranulation releases proteases, myeloperoxidases, and other cytotoxic mediators that can damage endothelial cells, impair myocardial function, and propagate systemic inflammation [42–44]. Our findings demonstrated associations between immature neutrophils and elevated circulating levels of lipocalin-2 and MPO, thus providing a potential mechanism for the role of immature neutrophils in the development of cardiac complications after SAH. Several studies previously demonstrated that both lipocalin-2 and MPO may directly contribute to the cardiac rhythm abnormalities, impaired cardiac function, and vascular disorders [45–49]. In parallel, elevated circulating lipocalin-2 and MPO were also associated with adverse cardiac events and worse overall outcomes. These observations provide convergent evidence that both quantitative (increased neutrophil burden) and qualitative (immaturity and degranulation phenotype) features of the neutrophil response are closely linked to the severity of cardiac stress and overall outcome after SAH.

A previous study demonstrated a significantly higher level of MPO in CD10^−^ immature versus CD10^+^ mature neutrophils [50]. In contrast, this study did not find significant differences in intracellular MPO levels, suggesting that MPO production and storage are not different between immature and mature neutrophils. One explanation for the observed association between CD10^−^ neutrophils and circulating MPO is potential differences in the control of degranulation between immature and mature neutrophils infiltrating the injured tissue. It is known that mature neutrophils have a higher capacity for phagocytosis compared to immature neutrophils [51,52], which are in part regulated by CD14 and CD16 [53,54]. Phagocytosis may reduce degranulation in mature neutrophils [55], thereby increasing the association between immaturity and degranulation. Immature neutrophils are also characterized by lower apoptosis compared to mature neutrophils [34,56]. Our studies revealed marginal but significant differences in the number of dead neutrophils, with fewer dead neutrophils in subjects with cardiac complications. It is plausible that immature neutrophils may also survive better after tissue infiltration, further contributing to the higher levels of MPO.

Taken together, these observations advance our understanding of the interplay between inflammation and cardiac stress following SAH. They support the concept of a brain-immune-heart axis, in which subarachnoid bleeding triggers sympathetic activation leading to immune activation, neutrophil mobilization, and both acute (sympathetic) and sub-acute (immune-mediated) myocardial injury. Future work should validate these findings in larger cohorts, investigate causal mechanisms linking neutrophils to electrophysiologic instability and myocardial dysfunction, and explore whether modulation of neutrophil maturation could reduce the burden of cardiac complications after SAH. Preclinical studies in animals with genetically altered granulocyte maturation are warranted. Finally, integrating these findings into multimodal models (neurologic, hemodynamic, immunologic) may improve the prediction and management of cardiac complications and, in turn, lead to better outcomes after SAH.

### Limitations

Several limitations should be noted. This was a single-center study, and although prospectively collected, the sample size was modest, which may limit generalizability. We relied on peripheral blood samples and could not directly assess immune activity in cardiac tissue or the central nervous system. ECG and echocardiogram data was obtained in a retrospective manner from chart review of hospital acquired studies and not in a timed sequence.

While associations were robust, our study design cannot establish causality; whether neutrophil maturation and activation directly contribute to myocardial injury or are bystander markers (for reasons including: systemic stress, secondary infection, or rate of antibiotic use) remain uncertain nor how this may be impacted by antibiotic administration. In this cohort, presumed or confirmed infection and antibiotic use were nearly universal and comparably distributed across cardiac and non-cardiac patient groups (74% vs. 69% presumed infection; 94% vs. 92% antibiotic exposure, respectively), which mitigates — though does not eliminate — the likelihood that these factors differentially account for the observed differences in immature neutrophil populations between groups. Nevertheless, the independent contribution of infection and antibiotic use to neutrophil dynamics cannot be fully disentangled in this observational dataset, and prospective studies with more granular infectious disease characterization will be needed to further clarify this relationship. Additionally, electrocardiographic measures such as QTc are influenced by medications and metabolic abnormalities, which we attempted to control for but cannot entirely exclude as confounders.

### Clinical implications and future directions

These findings have several potential clinical implications. First, assessment of neutrophil subspecies and inflammatory markers (including measures of neutrophil maturity and degranulation) might serve as early biomarkers to stratify patients by risk of cardiac dysfunction after SAH. This would guide monitoring (e.g., ECG frequency, telemetry), electrolyte and metabolic correction, and cautious use of QT-prolonging medications. Second, therapies that modulate neutrophil activation or degranulation may offer therapeutic benefit. For example, agents that inhibit neutrophil proteases, block excessive myeloperoxidase activity, or dampen emergency granulopoiesis might reduce myocardial injury or repolarization abnormalities. Third, serial monitoring of QTc in conjunction with immunologic profiling might help prognosticate and tailor therapeutic interventions —for example, identifying patients with rising neutrophils/immature neutrophils and prolonged QTc might trigger more aggressive supportive care or interventions aimed at limiting inflammation.

## Supplementary Information

Below is the link to the electronic supplementary material.

Supplementary Material 1

## Data Availability

This study was conducted under approval of the Institutional Review Board, and all participants (or their legally authorized representatives) provided written informed consent prior to enrollment. Biologic specimens were prospectively collected and processed according to the approved protocol, and clinical data were obtained through structured medical record review.The datasets generated and/or analyzed during the current study are not publicly available due to the inclusion of protected health information (PHI) and the ethical and legal restrictions imposed by the Health Insurance Portability and Accountability Act (HIPAA). These restrictions include direct and indirect identifiers such as dates of birth, medical record numbers, and dates of hospitalization.A de-identified dataset, with all direct identifiers and relevant indirect identifiers removed (including date of birth, medical record number, and hospitalization dates), is available from the corresponding author upon reasonable request and in accordance with institutional policies, applicable regulations, and, where required, a data use agreement.Investigators interested in accessing the data for reproducibility or secondary analyses are encouraged to contact the corresponding author.

## Declarations

### Data Sharing

This study was conducted under approval of the Institutional Review Board, and all participants (or their legally authorized representatives) provided written informed consent prior to enrollment. Biologic specimens were prospectively collected and processed according to the approved protocol, and clinical data were obtained through structured medical record review. The datasets generated and/or analyzed during the current study are not publicly available due to the inclusion of protected health information (PHI) and the ethical and legal restrictions imposed by the Health Insurance Portability and Accountability Act (HIPAA). These restrictions include direct and indirect identifiers such as dates of birth, medical record numbers, and dates of hospitalization. A de-identified dataset, with all direct identifiers and relevant indirect identifiers removed (including date of birth, medical record number, and hospitalization dates), is available from the corresponding author upon reasonable request and in accordance with institutional policies, applicable regulations, and, where required, a data use agreement. Investigators interested in accessing the data for reproducibility or secondary analyses are encouraged to contact the corresponding author.
