# Supplementary material for: Immature neutrophil response is associated with cardiac complications after non-traumatic subarachnoid hemorrhage
Source: Clin Exp Med. 2026 Jul 22;26(1):359. doi: 10.1007/s10238-026-02262-9 (PMC13677643; doi:10.1007/s10238-026-02262-9)
Supplement: Supplementary file 1 [file 10238_2026_2262_MOESM1_ESM.docx]

**Supplemental Table 1** Cardiac Abnormalities Score in study subject

**Supplemental Table 2**. Modified Poisson generalized linear regression for cardiac stress, accounting for covariates contributing to systemic inflammatory response at PBD1 and PBD3.
